# Commentary: What young people want from psychotherapy

**DOI:** 10.1111/papt.12542

**Published:** 2024-08-02

**Authors:** Kerry Gibson, Jessica Stubbing

**Affiliations:** ^1^ School of Psychology University of Auckland Auckland New Zealand; ^2^ Koi Tū: Centre for Informed Futures University of Auckland Auckland New Zealand

**Keywords:** adolescents, lived experience, psychotherapy effectiveness, youth mental health, youth‐informed interventions

## Abstract

In recent times there has been increasing acknowledgement of the importance of attending to the agenda of people with lived experience in psychotherapy research. In particular, young people's voices have been recognised as central to the design and development of psychotherapies that work for them. It is important to recognise the limits of professional agendas and make sure that young people's own priorities are represented in the indicators against which we measure change in research evaluations of psychotherapy. This requires an extension of evaluation research indicators from psychiatric symptomatology, to include aspects of wellbeing that matter to young people themselves. This article joins others in calling for a shift from the focus on symptom change in the evaluation of psychotherapy with youth, to acknowledge subjective indicators identified through research conducted with young people. New indicators might, for example, be centred on the degree to which young people experience increased capacity for acceptance of their emotions, a comfortable sense of identity, improved relational trust, and a stronger sense of their own agency. If psychotherapy is to be meaningful to young people, it is vital that we tailor it to young people's own needs and priorities and evaluate it against the aspects of change that matter to them.

## CONSTRAINTS ON WHAT COUNTS AS EFFECTIVE PSYCHOTHERAPY

The turn to an evidence‐based practice model in psychotherapy marked an important shift in service provision and has brought with it many benefits. However, it is time to re‐evaluate the constraints on what counts as evidence in the field of psychotherapy research evaluation, whose agendas these represent, and what voices and ideas might have been marginalised. Researchers and clinicians have historically shaped the focus of the evidence base for psychotherapies, but in recent times, there has been a growing recognition of the importance of including the views of those with lived experience of mental health problems. There is also increasing awareness of the ways in which young people's voices have been absent from consideration in the design of interventions aimed at addressing their mental health needs.

Despite some debate about what counts as evidence in psychotherapy, under the influence of evidential hierarchies used in other branches of health, randomised controlled trials or well‐controlled pre‐ and post‐cohort designs have long been the preferred method for evaluating the effectiveness of any psychotherapy approach (Spring, 2007). These kinds of research methods rely on the measurement of change using pre‐determined criteria that can, ideally, be attributed to the intervention. While in theory, the definition of these criteria is flexible, in practice they have largely followed a professional agenda framed by an emphasis on psychiatric diagnosis. This has resulted in research evaluation of any psychotherapy intervention being judged by the extent to which symptoms have reduced in severity. While this might indeed be one indicator of success, this approach has contributed to an illusion that symptoms are all that counts in evaluating the worth of any particular psychotherapy intervention. This has obscured the significance of other elements that might be central to psychotherapy effectiveness from the perspective of any particular client group.

However, recent years have seen promising shifts towards recognition of a broader range of indicators of effectiveness in psychotherapy research, and researchers are beginning, for example, to include measures of general functioning and quality of life in evaluation research (Knekt et al., 2015). There has also been particular recognition of the need to develop measures that are designed specifically for youth populations, and an awareness that these can contribute to the development of effective mental health services (Kwan & Rickwood, 2015; Rickwood et al., 2023; Thapa Bajgain et al., 2023). This is a good start, but we argue that more attention needs to be paid to the views and priorities of young people in determining what changes should be considered as indicators of an effective therapeutic approach.

The mental health professions have a long history of ignoring the voices of people who use their services (Fisher & Spiro, 2010). The constraints that excluded the voices of adults with mental health difficulties are exacerbated in relation to young people, who are often seen as incapable of commenting rationally on what they need (Zirkelback & Reese, 2010). Fortunately, there is now greater awareness of the contribution that young people with lived experience can make to mental health research, and the importance of ensuring that psychotherapy interventions fit with the priorities of those who use and potentially benefit from them (Simmons, 2021).

In clinical work, there have also been important shifts which recognise the value of systematically requesting client feedback and responding to this in psychotherapy (Miller et al., 2006). A goal‐based outcomes approach to evaluating psychotherapy with young people has now also gained considerable traction and respectability in clinical services. Tracking a young client's progress in psychotherapy against their own goals and priorities represents a profound shift in the approach to psychotherapy outcomes (Jacob et al., 2022; Jacob et al., 2023; Law & Jacob, 2013). Alongside this idiographic approach to evaluating client outcomes, it is however still important to develop population level indicators for the effectiveness of any psychotherapy for a particular population (Sales et al., 2023). Population level indicators allow us to recommend a general approach to intervention that might be more suitable for working with youth (in comparison, for example, to an approach that might work for adults or younger children). This focus is not intended to undermine the hard‐won recognition of the value of idiographic approaches to psychotherapy evaluation, and it would be important to retain this alongside a broader research agenda. One way of addressing this dilemma is to use research methods which can capture young people's definitions and priorities for their own mental health and build on these to inform indicators of psychotherapy effectiveness.

## WHAT DO YOUNG PEOPLE WANT FROM PSYCHOTHERAPY?

In recent years researchers have attempted to capture young people's own experiences, reflections, and priorities for mental health and professional support for this. This body of research suggests that young people might prioritise positive changes in their relationships, and the development of autonomy and identity alongside improvement in mood (Donald et al., 2018; Lavik et al., 2018; Mehta et al., 2023). We have conducted a series of studies in New Zealand over the past decade using open‐ended qualitative methods to explore young people's engagement with mental health support. The collective weight of this work suggests four indicators that fit closely with current youth priorities (Gibson, 2022).

### Acceptance of emotion

One of the greatest concerns that young people have expressed is that their distress is illegitimate and that they will be judged negatively by others. They worry that their problems will be dismissed by professionals as a product of the turmoil of youth, and that the reasons they give for their distress will be minimised. Alternatively, they fear that their problems will be pathologised and stigmatised. One of the markers for successful psychotherapy is that it provides a non‐judgemental validation of their experience of distress. This includes being listened to respectfully, being taken seriously, and having their accounts believed. Validation has been recognised as one of the most powerful tools in psychological intervention, leading to increased self‐acceptance and improved emotional regulation (Koerner & Linehan, 2004). Effectiveness indicators suggested by this set of priorities might include changes in the extent to which a young person experiences their emotions as acceptable and believes that these can be understood by others.

### Developing a comfortable identity

It has long been recognised that identity‐making is a key developmental task for young people (Arnett, 2007; Erikson, 1994). This is even more pronounced for current generations who negotiate a broader array of identity options. Unsurprisingly, young people see a helpful psychotherapy as one which supports the development of their sense of self. For young people, psychotherapy works when it is adapted to their individuality, allows them to explore who they are, and to establish a strong sense of identity. Having a coherent identity contributes to wellbeing and provides young people with a sense of their own worth and purpose (Crocetti, 2017). This suggests the value of effectiveness indicators which capture any improvement in the extent to which a young person feels comfortable with their identity and can conceive of a future guided by their own strengths and values.

### Having good relationships

Relationships are very important to young people. Difficult relationships are often a major contributor to mental health distress and positive relationships reflect wellness (Oldfield et al., 2016). Understandably, young people see helpful psychotherapy as one where they have built a strong relationship with a clinician, a factor well‐recognised in the psychotherapy literature (Lambert & Barley, 2001). This relationship does not simply provide a medium that enables other aspects of the psychotherapy to be delivered effectively, for young people, it is the relationship itself that is seen to be of benefit. When young people experience a meaningful connection with a clinician in psychotherapy, this leaves them with a sense that relationships can be trusted, that they are worthy of care, and that they are valued by others. Indicators of effectiveness which assess the extent to which a young person feels they can be part of good quality, reciprocal relationships with others are likely to be helpful for measuring the success of a psychotherapy intervention.

### Experiencing agency

Young people, relative to adults, experience less power and are acutely aware of constraints on their agency. They look to psychotherapy to provide them with space to articulate their own views and to exercise their own choices. This echoes the centrality that client‐agency has been given in some accounts of psychotherapy (Williams & Levitt, 2007). Young people appear to evaluate the success of psychotherapy as the extent to which it empowers them and gives them a sense that they can take control of their lives in meaningful ways. This points towards indicators of effectiveness which examine shifts in a young person's sense of agency and felt ability to exercise appropriate control over their lives.

## CONCLUSIONS

While there have been significant strides in recognising the value of including young clients' views within clinical practice, research evaluations of psychotherapy still tend to prioritise a narrow set of indicators of effectiveness dominated by professional agendas. There is a need to establish the value of indicators that more closely align with young people's own priorities. Many clinicians working with young people in mental health settings will recognise the salience of acceptance, identity, relationships, and agency in their work. Our research with young people suggests these are the issues that are indeed important for young people. The four factors identified in this commentary can inform indicators of effectiveness in psychotherapy research that more accurately match the priorities of this population and might be useful in informing the generation of research measures or directing the focus of qualitative research. This youth‐informed approach to the evaluation of psychotherapy can sit usefully alongside the advantages of an idiographic approach that recognises the uniqueness of each individual's psychotherapy experience (Sales et al., 2023).

Capturing the voices of young people who experience mental health problems is more than a moral issue; more than a question of righting the balance of power. It is a matter of ensuring that we are really using the full range of evidence to inform our clinical work. It is important, however, that we begin this journey by challenging the idea that the agenda on outcome criteria for psychotherapy needs to be set by professionals and opening up to the possibility of other options informed by young people's lived experience of mental health problems and service use. This requires us to question what we have historically considered ‘evidence’ as we have defined evidence‐based practice in research to date.

There is no doubt much more to be learned about the kind of outcomes young people might be looking for in a successful psychotherapy intervention and how researchers can meaningfully evaluate effectiveness within these four indicators, and there are likely to be differences for diverse groups of young people yet to be discerned. There is also potential to build on the synergies with a goal‐based approach to clinical outcomes, to explore common themes in the goals that young people identify and therefore how we might define youth‐informed indicators of effectiveness in research (Sales et al., 2023).

## AUTHOR CONTRIBUTIONS

**Kerry Gibson:** Conceptualization; writing – original draft; writing – review and editing. **Jessica Stubbing:** Conceptualization; writing – original draft; writing – review and editing.

## CONFLICT OF INTEREST STATEMENT

The authors have no conflict of interest to declare in relation to this article.

## Data Availability

There is no data vailable for this commentary.
